# Spectacular Improvement of Paradoxical Reaction in Tuberculosis after Tumor Necrosis Factor-Alpha Antagonist Therapy

**DOI:** 10.7759/cureus.50596

**Published:** 2023-12-15

**Authors:** Myriam Samad, Charles Antoine Dallevet, Yacine Tandjaoui-Lambiotte, Anne Bourgarit, Pierre Jaquet

**Affiliations:** 1 Pulmonology & Infectious Diseases Department, Delafontaine Hospital, Saint-Denis, FRA; 2 Intensive Care Unit, Delafontaine Hospital, Saint-Denis, FRA; 3 Internal Medicine, Jean Verdier Hospital AP-HP, Bondy, FRA

**Keywords:** tnf-alpha antagonist, disseminated miliary tuberculosis, neurotuberculosis, paradoxical reaction, corticotherapy, medical intensive care unit (micu)

## Abstract

We report the case of a 42-year-old immunocompetent Indian patient presenting with miliary tuberculosis complicated by respiratory failure requiring intubation. Conventional quadritherapy was initiated for wild-type *Mycobacterium tuberculosis*. On day 29 of antibiotic treatment, persistent fever and neurological deterioration prompted the diagnosis of multiple brain and medullary tuberculomas, some surrounded by edema. Laboratory investigations ruled out meningitis and subtherapeutic drug concentrations. To enhance cerebrospinal fluid penetration, ethambutol was replaced with levofloxacin on day 30, and rifampicin doses were increased to 30 mg/kg. Dexamethasone was introduced on day 30 to address the paradoxical response to antituberculosis therapy, but neurological deterioration persisted, leading to hemiparesis and coma, with concurrent development of acute respiratory distress syndrome. As salvage therapy, an anti-tumor necrosis factor agent, infliximab (IFX), was administered on day 40. Rapid clinical improvement was observed, marked by awakening and subsequent weaning from respiratory ventilation just eight days after the first IFX infusion. The patient was discharged from the intensive care unit 10 days post-IFX initiation, with steroids discontinued one month after IFX introduction. Both antituberculosis treatment and IFX infusions (seven in total) were maintained for one year. Clinical and radiological evaluation at one year demonstrated complete clinical and radiological recovery.

## Introduction

Tuberculosis is a major health problem, with 10.6 million new cases in 2021 according to the Global Tuberculosis Report 2022. Paradoxical deterioration upon tuberculosis chemotherapy, known as tuberculosis-immune reconstitution inflammatory syndrome (TB-IRIS), is a well-described entity in human immunodeficiency virus (HIV) patients after receiving antiretroviral therapy (ART) [1]. The pathology of TB-IRIS is attributed to cytokine release, specifically that of interleukin 6 and tumor necrosis factor (TNF) [2]. A similar reaction exists in non-HIV patients, first described in 1954 [3]. Paradoxical reaction (PR) is frequent in non-HIV extrapulmonary tuberculosis, occurring in 20%-25% of patients, and has an excellent prognosis except in central nervous system (CNS) involvement [3,4]. Pathogenesis is possibly secondary hypersensitivity to tuberculoprotein and a decrease in immune suppression [5,6].

PR/IRIS is a diagnosis of exclusion, requiring the elimination of drug toxicity or reaction, tuberculosis treatment failure caused by resistance, another infection, and poor adherence to treatment [5]. Indication for therapy depends on both severity and presentation. Corticosteroid therapy has proven effective in the context of constrictive pericarditis and meningitis and for the prevention of TB-IRIS in high-risk patients upon ART treatment [7-9]. In the absence of life-threatening conditions, corticosteroids have been anecdotally used to relieve symptoms and nodes or abscess compression, but with a lack of controlled trials [10-12].

Anti-TNF is a potential treatment in cases of PR/IRIS resistant to corticosteroids, especially in CNS involvement. In a recent review of the usage of anti-TNF in PR/IRIS, 24 cases were identified. Despite being used as a second-line treatment and as a salvage therapy in severe cases, the rate of improvement was 100% [13]. Here, we present a case report emphasizing the potential benefit of TNF antagonism in PR.

## Case presentation

A 42-year-old male of Indian origin, living in France since 2002, presented to our hospital with a one-month history of general status alteration. He reported asthenia and 19 kg of unintentional weight loss during an interval of less than six months. The patient sought medical care due to the appearance of shortness of breath graded on the modified Medical Research Council Dyspnea Scale. He denied experiencing night sweats, fever, cough, gastrointestinal, or genitourinary symptoms. The patient was not known to be immunocompromised, with negative HIV results, no hypogammaglobulin levels, and no history of immunosuppressive therapy or recurrent infections. No known past medical history was reported for tuberculosis or screening upon his arrival to France for latent tuberculosis.

Upon arrival at the emergency department, the patient exhibited a heart rate of 143 beats per minute, an oxygen saturation of 88% on room air, a respiratory rate of 40 breaths per minute, and a temperature of 37.8°C. Physical examination revealed a normal neurological status, no palpable adenomegaly, no signs of hypoperfusion, regular heart sounds with no added sounds, bilateral basal crepitation on lung auscultation, a soft non-tender abdomen, and no hepatomegaly or splenomegaly. An electrocardiogram showed sinus tachycardia. Laboratory findings ruled out severe acute respiratory syndrome coronavirus 2, influenzae, respiratory syncytial virus, and urinary tract infection. However, elevated C-reactive protein levels were noted (50 mg/L), along with the absence of leukocytosis and an increased D-dimer concentration (6,535 ng/mL).

**Figure 1:**
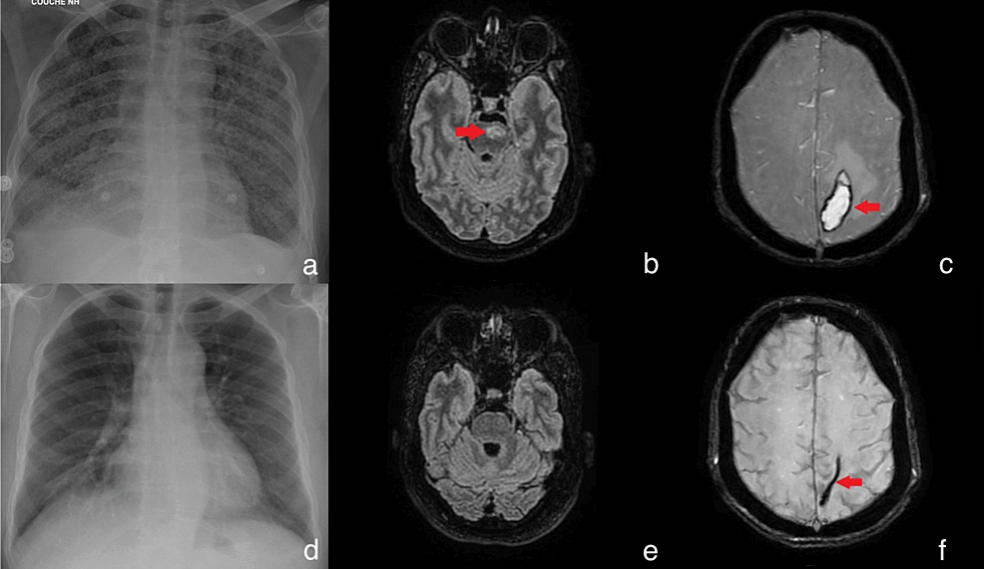
Evolution of intracranial and thoracic findings: a year-long imaging assessment. Imaging at baseline: a. Chest X-ray reveals numerous bilateral small opacities resembling millet seeds. b. Cerebral magnetic resonance imaging displays left median and paramedian pontine lesions. c. Left parietal hematoma observed with perilesional edema. Imaging after a year: d. Resolution of miliary opacity. e. Disappearance of pontine lesions observed. f. Residual sequelae of the parietal hematoma and resolution of its perilesional edema.

A chest X-ray revealed diffuse bilateral opacity (Figure 1, Panel a). A computed tomography pulmonary angiogram confirmed the presence of diffuse micronodules of hematogenous spread, compatible with military tuberculosis, along with bilateral small pleural effusion. The patient was administered oxygen (6 L/minute) and transferred to the pulmonary and infectious disease floor.

On day two, the patient underwent a bronchoscopy for microbiological documentation. The procedure was performed in the intensive care unit (ICU) for monitoring due to his critical condition. Unfortunately, respiratory failure progressed during the intervention, followed by shock. As a result, the patient was rapidly sedated and intubated. The relevant arterial blood gas parameters were a lactate level of 7.8 mmol/L, a pH of 7.24, a PaO_2_/FiO_2_ ratio of 111, and a positive end-expiratory pressure of 6 cmH_2_O.

The patient was placed on noradrenaline and received antibiotherapy with spiramycin, cefotaxime, linezolid, and amikacin. To enhance diagnostic yield, a lumbar puncture and a transjugular hepatic biopsy were conducted. The lumbar puncture ruled out meningitis, revealing cytology with a red blood cell count of 39/mm^3^ and leukocytes of 4/mm^3^ (no differential was performed). Glucose levels were 4.7 mmol/L, protein levels were elevated at 0.7 mmol/L, and both polymerase chain reaction (PCR) and culture results were negative. The patient’s urine direct exam was negative, but cultures returned positive after 30 hours. The hepatic biopsy revealed a non-caseating granuloma on histopathology. A workup for acquired immunodepression showed negative serology for HIV.

On day three, the PCR results from bronchoalveolar lavage returned positive for tuberculosis with an absence of mutation associated with resistance to rifampicin of the *rpoB* gene and isoniazid of the *KatG* and *InhA *genes, whereas the lumbar puncture remained negative for tuberculosis. A quadritherapy regimen with intravenous (IV) rifampicin 600 mg (10 mg/kg), isoniazid 200 mg (3.5 mg/kg), ethambutol 1,200 mg (20 mg/kg), and per os pyrazinamide 1,750 mg (30 mg/kg) was initiated. The antibiotherapy was de-escalated to cefazoline due to the identification of methicillin-resistant *Staphylococcus aureus* as a probable bacterial superinfection. Adjunct corticosteroid therapy with prednisolone 1 mg/kg was considered necessary due to significant inflammation.

The patient’s ICU stay was further complicated by ventilator-associated pneumonia due to *Pseudomonas*, drug reaction to ceftazidime, volume overload, and pneumothorax. These complications necessitated adjustments in antibiotherapy, continuous renal replacement therapy, and chest tube insertion with drainage. The patient showed improvement in his ventilator parameters, with an FiO_2_% of 25% and a PaO_2_/FiO_2_ ratio of 305, and weaning from vasopressors.

On day 29, following sedation withdrawal, the patient exhibited left hemiparesis and hyperactive deep tendon reflexes. Neuroimaging via brain and medullary magnetic resonance imaging (MRI) revealed multiple lesions compatible with tuberculoma, along with a left parietal hematoma surrounded by edema, moderate hydrocephalus with signs of resorption, arachnoiditis, and lumbar epiduritis (Figure 1, Panels b and c). A lumbar puncture indicated moderate intracranial hypertension with a pressure of 24 cmH_2_O. PCR testing of his tuberculosis cerebrospinal fluid (CSF) remained negative, and a fundoscopic exam showed no papilledema. Urgent CSF shunt surgery was not indicated by neurosurgeons. The rifampicin dose was increased to 20 mg/kg/day to optimize pharmacodynamic targeting in the CSF. Corticosteroids were reintroduced, this time with dexamethasone 25 mg.

An expert opinion was obtained from the Centre National de Référence des Mycobactéries, suggesting further optimization of the tuberculosis therapy. Rifampicin doses were increased to 1,800 mg (30 mg/kg), isoniazid to 300 mg (5 mg/kg), and ethambutol was switched to levofloxacin 500 mg due to better CSF penetration, with a CSF-to-serum ratio of the area under the curve of approximately 1. Additionally, infliximab 300 mg was initiated initially every 15 days as a treatment for a probable PR. Prophylaxis for opportunistic infection with atovaquone was also added.

Anti-TNF therapy (infliximab) was administered after 10 days of intensifying antibiotherapy due to persistent neurological symptoms. The patient’s subsequent evolution was favorable, with successful weaning from mechanical ventilation on day 48, leading to a transfer to the regular floor after approximately two months.

On the regular floor, the rifampicin dose was reduced to 20 mg/kg on day 27 of therapy, and the route of administration was switched to oral. Weaning of steroids was initiated after one month of therapy, while infliximab was continued as maintenance immunosuppressive therapy. A brain MRI performed on the ninth week of antituberculosis treatment showed regression of the hematoma and decreased contrast enhancement.

After three months of hospitalization, the patient was discharged to a rehabilitation center where he underwent physiotherapy while maintaining antituberculosis therapy and infliximab perfusions. Regular follow-up consultations were conducted to monitor for PR relapse. The intensive phase of antituberculosis therapy was extended to six months (isoniazid/rifampicin/pyrazinamide/levofloxacin), followed by six additional months of isoniazid/rifampicin during the continuation phase. The total duration of antituberculosis therapy was one year, given in association with infliximab. The last consultation, conducted after a year of therapy, revealed that the patient had no neurological or respiratory symptoms. The follow-up imaging revealed the resolution of the miliary pattern on the chest X-ray (Figure 1d), the complete disappearance of multiple intracranial tuberculomas, and the chronic hematoma on the MRI (Figure 1, Panels e and f).

## Discussion

The patient was initially treated for acute miliary tuberculosis complicated by multiorgan system failure (i.e., shock, respiratory, and renal failure). The initial sites of involvement were the lungs, lymphatic system, liver, and urinary system. Brain extension was not explored at that time. At presentation, the patient had no neurological manifestation, which was first noted after one month of treatment. Although the paradoxical progression of the intracranial tuberculoma on antituberculosis treatment could not be well evaluated due to the absence of an anterior exam, we think that the patient could fall into this category because of the absence of neurological manifestation at presentation and its appearance on antituberculosis treatment.

Despite intensified tuberculosis therapy and the reintroduction of corticosteroids, there was no improvement in neurological symptoms. Anti-TNF therapy (infliximab) was initiated on day 10, leading to positive progression, extubation, and transfer to the regular floor after two months.

Whether it was a worsening of a preexisting lesion or an appearance of a new one, this progression could not be explained by a subtherapeutic treatment due to close surveillance of the pharmacodynamics of antituberculosis treatment, nor by resistance, as the organism was sensitive. In several studies on this form of PR, surgery had no role, and prolonged antituberculosis therapy along with corticosteroids was the standard of care [14,15]. Unfortunately, our patient did not improve on the same standard of care. Here, the significance of anti-TNF therapy comes into play as salvage therapy. In a literature review conducted by Armange et al., all 24 patients on TNF-α antagonists showed improvement [13]. Anti-TNF therapy is considered a safe and effective salvage therapy in severe PR.

## Conclusions

PRs warrant close attention in patients presenting with neurological manifestations during tuberculosis treatment. Anti-TNF therapy has emerged as a promising salvage option in severe cases of PR. The potential benefits of utilizing anti-TNF therapy as a first-line treatment or as a corticoid-sparing modality should be carefully evaluated to tailor therapies effectively for these patients.
